# Gender bias in the Erasmus network of universities

**DOI:** 10.1007/s41109-020-00297-9

**Published:** 2020-09-15

**Authors:** Luca De Benedictis, Silvia Leoni

**Affiliations:** 1 Department of Economics and Law, University of Macerata, Via Crescimbeni 14, Macerata, 62100 Italy; 2 Department of Business and Management, Luiss, Viale Romania 32, Rome, 00197 Italy; 3 Department of Economics and Social Sciences, Marche Polytechnic University, Piazzale Martelli 8, Ancona, 60121 Italy

**Keywords:** Erasmus, Gender bias, Network Analysis, Degree distribution

## Abstract

The Erasmus Program (EuRopean community Action Scheme for the Mobility of University Students), the most important student exchange program in the world, financed by the European Union and started in 1987, is characterized by a strong gender bias. Female students participate to the program more than male students. This work quantifies the gender bias in the Erasmus program between 2008 and 2013, using novel data at the university level. It describes the structure of the program in great detail, carrying out the analysis across fields of study, and identifies key universities as senders and receivers. In addition, it tests the difference in the degree distribution of the Erasmus network along time and between genders, giving evidence of a greater density in the female Erasmus network with respect to the one of the male Erasmus network.

## Introduction and prior research on the Erasmus program

At its 30^th^ anniversary celebrations in 2017, the Erasmus program counted more than 9 million participants since its creation, increased to more than 10 million in 2018. The program, which allows its participants to study or take an internship in a foreign country,^1^ has become very popular among university students whose participation is increasing year after year. Its popularity has made it a true cultural phenomenon, and, given the successful outcome of Erasmus+, the European Commission has proposed, for the 2021–2027 plan, to double the funds allocated to the program in order to support the mobility of 12 million people, making the program more inclusive, allowing the participation of those coming from disadvantaged families background and less inclined to international mobility.^2^ With its objective of inclusion, the program has also become a cornerstone for equal opportunities, with many of its projects, for example, directly aimed at the promotion of gender equality. Nevertheless, the participation in the Erasmus program itself is characterized by a remarkable and long-lasting gender bias, that favors women, since its launch.

Evidence shows that the number of women participating in the program has been higher than that of men up to the 1990s (Maiworm 2001). This gender gap is reported by Bottcher et al. (2016) for the academic year 2011-12, across both countries and subject area.^3^ More broadly, the evidence of a bias in favor of female students in the Erasmus program can be related to the research suggesting that women participation in global academic mobility is large due to the growing number of women enrolled in higher education, as well as the greater gender parity across the world (Bhandari 2017). Globally, this pro-female trend may be attributed also to the opportunities provided by targeted scholarship and fellowship programs for under-represented groups to pursue advanced study outside their home countries.^4^ Nevertheless, under-representation of women among inbound international higher education students is highly significant in some countries, as in the United States, where although the progress made in the last decades, the gender imbalance in terms of incoming international students is still widening, possibly because of the rising number of international students in STEM-related^5^ fields which are typically dominated by male students (Bhandari 2017; Myers and Griffin 2019). The United Kingdom, instead, hosts more female international students rather than male students (Myers and Griffin 2019), and Faggian et al. (2007) also show evidence that female graduates from the U.K. are more mobile than men graduates. The authors rationalize this outcome by suggesting that women use migration as a form of compensation for gender discrimination in accessing the local labor market when foreign labor markets offer better job opportunities. The issue of student international mobility also nicely overlaps with the recent research on temporary migration and commuting, as emphasized by Monte et al. (2018).

This work solely focuses on Erasmus mobility for study reasons. The aim is to analyze the gender difference in the participation in the program and its variation over the years. We use the data available on the EU Open Data Portal, which correspond to six datasets, describing students flows between universities in European countries since the academic year 2008/09 until 2013/14. We compare the extremes of this time interval in most of the analysis.

First of all, the paper provides a descriptive analysis of gender bias by field of study, comparing 2008/09 as the initial year and 2013/14 as final year. The fields of study are also aggregated in STEM and non STEM disciplines to further highlight the differences discovered. This analysis is performed at country level. We find that the STEM disciplines are characterized by a male prevalence in mobility participation; the opposite can be observed for the non-STEM sectors. In general, from 2008 to 2013 we observe a trend towards parity in gender and changes towards this tendency happened in particular in Eastern and some Mediterranean countries.

Secondly, the paper explores the network of universities participating in the Erasmus program and offers a comparison between the years 2008 and 2013. The use of network analysis, specifically focusing on gender bias, allows to study the Erasmus weighted directed network by gender, and allows to identify the most important universities hosting and sending female and male students abroad. We find evidence of gender bias in favor of women, with a ratio between female and male links equal to 1.338 in 2008 and 1.329 in 2013, showing an overall persistence of the bias. Compatibly, the graph of female flows presents a higher density than the one of male flows, with a higher number of both active nodes and arcs. Considering the incoming flows of students, the most central node in the female graph is the Universidad de Granada, in Spain, confirming its centrality in both years considered; in the graph of male flows, the Universitat Politecnica de Valencia is the most central in 2008, while the top position is reached by the Universidad de Granada in 2013. Considering the outdegree centrality, the Univerzita Karlova of Prague and the Universidad de Granada are the most central in 2008 respectively for the male and female flows, while again the Universidad de Granada is on top of both ranks in 2013. Further gender differences are made evident through the analysis: almost half of the university exchanges are gender specific, involving only males or only females, the level of inter-gender bilateral university flows is around 50%; the network of female flows tends to be characterized by a higher level of reciprocity and homophily; the female Erasmus network presents a lower number of isolated universities and higher connectivity; the female network also shows a higher prevalence of sending universities over receiving universities, unlike the male one.

Thirdly, gender imbalance is further studied in terms of the degree distribution of the Erasmus network. Considering the directed and unweighted Erasmus network of universities, the analysis explores the possible changes in the indegree and outdegree distributions along time and between gender and tests a power law fitted model to the data. Our findings differ from what found by Derzsi et al. (2011), which conclude that the degree distribution of the Erasmus network in 2003 follows an exponential distributional model, although in that work the authors study the overall distribution without differentiating for gender. The results of this paper suggest that the indegree distribution has an opposite behavior along time in the tail of the female and male distribution. The former seems not well represented anymore by a power law model in 2013 as it was in 2008; in the latter, instead, the tail appears well described by a power law model. Our interpretation is that in the case of the indegree male distribution, the top universities, in terms of connections, are moving away from the body of the distribution, while the opposite is happening for the female, meaning that passing from 2008 to 2013 the universities with the most male links have been growing this kind of connection, while the universities with the most female connections are reducing their difference with the bulk of the distribution, overall resulting in a tendency towards balancing the initial disparity in favor of women.

As it is evident, our study is not the first one to apply Network Analysis to the flow of Erasmus students. Several studies opened the way to the methodology we adopted. Erasmus student mobility among European Higher Education institutions is also analyzed in Breznik (2017) solely for Engineering students between 2007 and 2013. The study recognizes the Spanish universities as those with the highest mobility of Engineering students and points out the top universities in terms of student exchanges. A similar application can be found in Breznik and Djaković (2016) which analyze the mobility related to Slovenian universities in the years 2007/08 and 2011/12 and highlight the most attractive countries and universities for Slovenian students as well as the top destinations for Erasmus students in Slovenia. The same approach is extended by Breznik and Skrbinjek (2020) with an analysis of hubs and authorities focusing on the countries participating in the Erasmus program. The authors provide a general overview of Erasmus mobility trends, revealing 3 different groups of countries: good senders and receivers, good senders only, and good receivers only. The hubs and authorities technique is further extended in Restaino et al. (2020) which in addition adopt a blockmodeling approach on the network of countries involved in the Erasmus mobility both for study and internship abroad. The authors provide also an analysis of homophily behaviors among countries and exploit educational indicators from Eurostat to include investments in higher education. Results reveal the presence of a core-periphery structure in the student mobility network, where key elements to attractiveness are economic benefits and investments in education. Besides the network approach, these works adopt the same dataset exploited in this paper. Finally, a global rather than European focus is adopted in Shields (2013) which applies Network Analysis to international student mobility data covering the period from 1999 to 2008, in order to better understand the globalization of education. The author seeks to study changes in the network rather than the network properties. His findings suggest that the network of international students has become more centralized, less densely connected and it shows structural similarities with the networks of world trade and world polity.

The main novelty of the present paper resides in i) the analysis of the gender gap in Erasmus flows. The literature focusing on this aspect in fact is still quite scarce. If the issue of gender bias has been faced often in the context of international student mobility, to the best of our knowledge, when considering the specific context of Erasmus mobility, gender differences have not been thoroughly studied, with the exception of Bottcher et al. (2016), whose findings about female over-representation in the program are in line with the results of this paper. Our contribution aims at filling this gap and, in addition, ii) it uniquely combines the analysis by gender, by field and at the university level rather than at country level, as most preferred in the extant literature (Restaino et al. 2020; Bottcher et al. 2016; Breznik and Skrbinjek 2020).

The paper is organized as follows: the first section provides a description of the data used in the analysis; the second section quantifies the Erasmus program general trend and gender imbalance in student flows across fields of study. In this section, the comparison between STEM and non STEM sectors can be found, as well as considerations about the overall trend towards gender parity and the changes seen in Eastern and some Mediterranean counties. The third section analyses the network of universities participating in the program, by gender. It shows strong gender bias in favor of female students, that persisted over the years considered. The section provides a set of centrality measures in the network, its density and highlights the top universities for sending and receiving students. The fourth section compares the network indegree and outdegree distributions over time and between genders, it highlights the change in the distributional model occurred over the years considered and tests for the best model to fit the empirical distribution; finally, the conclusive section summarizes the results of the analysis, draws some final observations regarding the possible future evolution of the gender bias in the Erasmus program and provides some suggestions for future research.

## Data

**Fig. 1:**
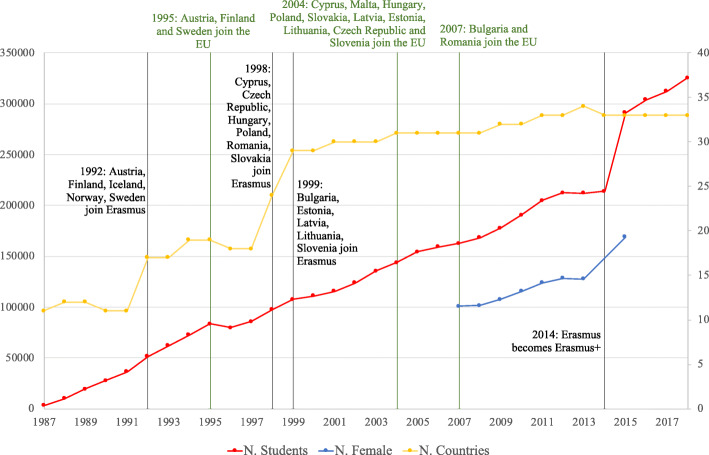
The history of Erasmus program from 1987 to 2018. The evolution of the Erasmus program is represented in terms of countries and students on mobility only for study reasons. The red line represents the number of mobile university students (left scale), the blue line represents the number of female university students on mobility only for the years in which the information is available (left scale), the yellow line represents the number of countries participating in the mobility (right scale). The grey vertical lines indicate the adhesion of new countries to the Erasmus program and the transformation of the program into Erasmus+. The green vertical lines identify some enlargements of the EU. Note that the value for the year 2014 is not an *ex post* figure, but a projection (European Commission 2015). Data on the number of participating countries and students are extracted from the Erasmus annual reports (European Commission 2000); information on female participants can only be derived for the period 2008–2013 and the year 2015

**Fig. 2:**
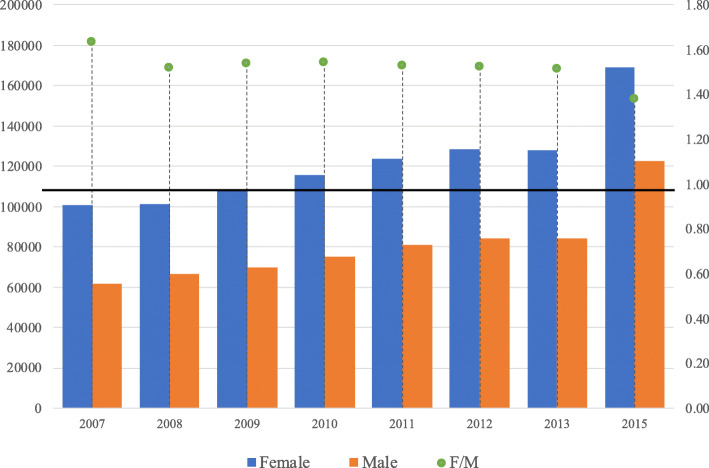
Gender balance in the Erasmus mobility over selected years. The blue bars indicate the number of female Erasmus students, the orange bars indicate the number of male Erasmus students (both to be read on the left vertical axis). The green indicator represents the value of the female over male (*F*/*M*) ratio (right vertical axis). The horizontal line positioned at the ordinate value of 1 on the right vertical axis corresponds to *F*/*M*=1. Information shown is based on the micro-data available on the EU Open Data Portal for the period 2008–2013 and on extrapolation from the Erasmus annual reports (European Commission 2000) for the other years represented (2007 and 2015). Information on gender participation in 2014 could not be retrieved

Data used in the analysis are available at the EU open data portal and are freely accessible. They consist of 6 datasets corresponding to the academic years from 2008/09 to 2013/14 and they contain observations for each participant to the mobility in the relative academic year including information on the type of mobility (study or placement), the home country and the host country, the home university and the host university, the field of study coded in the ISCED^6^ 1997–2011 classification (UIS 2012), the participant gender, the level of study (first or second cycle), the duration of the mobility, the amount of the grant received and the language used in the mobility. Although the Erasmus program has a long history, micro-data gathered in this format and containing the information described are not available for any further year. For this reason, since our analysis requires information about the field of study, and the unit of analysis chosen for the network analysis is the institutional level, thus universities, the study is limited to the period from the academic year 2008/09 to 2013/14. For simplicity, the reference years will be abbreviated as 2008 and 2013 respectively. Data have been aggregated at country level exclusively for the analysis of gender by field of study (The Erasmus program section). To consider variations across the period 2008–2013, each analysis performed compares the initial year (2008) with the final year (2013). In order to have an overview on the evolution of the program and gender participation, as shown in Figs. 1 and 2, additional information at country level has been retrieved from the annual reports by the European Commission (2000).

From the 6 datasets available, we only consider observations related to study mobility type and clean the data from misclassification, mismatched coding and foreign names, and the use of specific Unicode characters present in the original datasets.

By counting the frequencies of unique connections between pairs of universities, we specify the weight of each connection, that represents the students flow, i.e. the number of students going on mobility from the sending university to the receiving university. Data are therefore collapsed so to have observations for each dyad of universities linked by at least one student in mobility, by gender and by field of study. The fields of study are grouped in macro fields according to the ISCED-F 2013 coding system (UIS 2014), which identifies 12 macro fields. Eventually, for the whole period of time considered, the datasets count 762304 observations in total, with 3148 universities present in the time span considered.

## The Erasmus program

Erasmus stands for European Region Action Scheme for the Mobility of University.^7^ It is a student mobility program created by the European Union in 1987. The program started with the idea of allowing European university students to study abroad in a European university, with the legal recognition of the mobility in the home university and providing a scholarship to cover the additional cost for studying in another country of the EU for a period of between three months and one year. The work that led to the official approval of the program saw the involvement of universities from all over Europe in order to establish the legal and financial basis necessary to develop and manage organizational and educational cooperation between universities underpinning the Erasmus program (Corradi 2015).

The objectives of Erasmus+ and the original Erasmus program can be summarised in strengthening the European identity, increasing individual skills, and, thus, their employability. By creating opportunities for study, training, work experience, and volunteering abroad, Erasmus aims to respond to the problems of unemployment and skills shortages in Europe and to modernize education and training systems. With respect to its objectives, the literature has highlighted the positive impact of Erasmus and its effectiveness both in terms of European identity and likelihood to find a job after graduation (Oborune 2013; Jacobone and Moro 2015; Engel 2010; Parey and Waldinger 2011; Bryła 2015).^8^ In addition, the Erasmus program is considered a successful example of European integration and a symbol of the construction of European identity.

### General trend

Over time, Erasmus has become an essential part of the unified European mobility programs in the area of education Socrates I (1994–1999), Socrates II (2000–2006) and Lifelong Learning Program (2007–2013) and has grown in size. The program started in 1987 with 11 participating countries and 3244 students on mobility and reached 33 participating countries in 2018 and 325495 university students on mobility for study reason (see Fig. 1).

The growth in the number of participating students has followed the growing trend of the countries participating in the program, which in many cases joined Erasmus before becoming EU Member States.^9^ The number of students participating in the mobility saw an unprecedented increase in 2015, going from 213879 to 291383, although the number of participating countries remained almost unchanged. In 2014 the program became Erasmus+ and changed its structure (with the EU Regulation 1288/2013): it is no longer exclusively dedicated to education, but also to training, youth and sport, and it no longer restricts participation to university students only, but also admits, for example, school and university teaching staff, as well as administrative staff. Therefore, it is an integrated program that has incorporated all the funding mechanisms for school and university student mobility implemented by the European Union until 2013 (e.g. Comenius, Leonardo Da Vinci and others). In 2013 the program reaches the largest number of participants, 34, the 28 EU Member States plus Switzerland, Iceland, Liechtenstein, Norway, North Macedonia, and Turkey. Since 2014, Switzerland no longer enjoys the status of participant to the program, but it is now a partner country, i.e. it has adopted a transitional solution financed with Swiss funds which still allows Swiss people and institutions to take part in the program. In 2019, participant countries have been 34 again with the official entry of Serbia. €14.7 billion were allocated to the Erasmus budget for the period 2014–2020, 40% more than the previous programming period, and, as already highlighted, for the period 2021–2027 the European Commission has proposed to double the figure to €30 billion (European Commission 2018). The program continues to grow with the aim of becoming more powerful and inclusive.

### Gender balance

Figure 2 shows the amount of female and male participants in the Erasmus program for some selected years (2007, the academic years from 2008/09 to 2013/14, and 2015: data on the first and the last year come from the Erasmus annual reports provided by the European Commission (2000) and the relative ratio between female and male students. The number of both female and male students grows following the growing general trend in participation seen in Fig. 1, with females representing a large majority for each year. Nevertheless, the ratio *F*/*M* slightly decreases over the years considered, signaling wider participation of men (see Fig. 2).

**Fig. 3:**
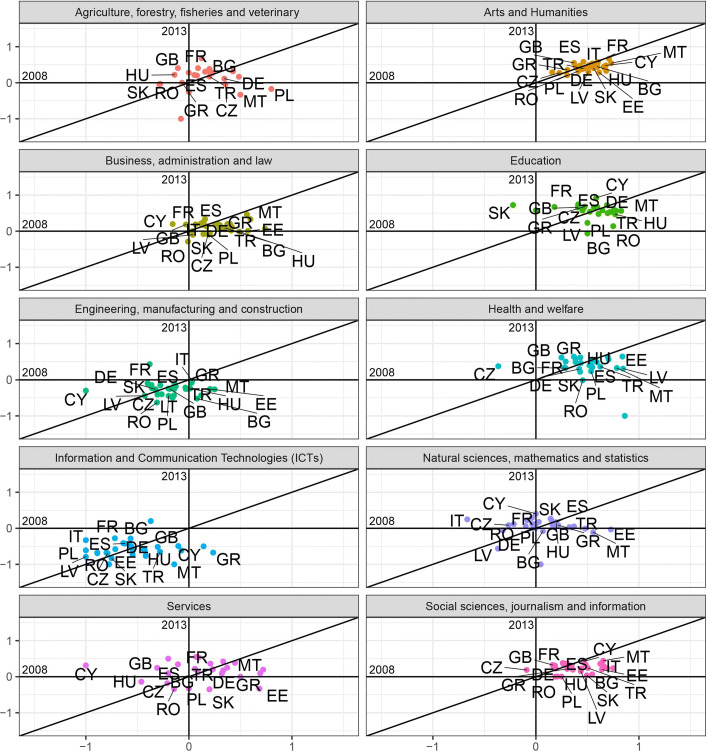
Gender balance in the incoming flows across fields of study for the year 2008 against 2013. The scatter plots show the *F*/*M*^*B*^ measure for the incoming flows of students for the years 2008 and 2013 for ten fields of study corresponding to the ISCED-F 2013 classification. Two macro fields (*Not known or unspecified* and *Generic programs and qualifications*) are not showed. The label of the following countries are not displayed to improve readability: Austria (AT), Belgium (BE), Croatia (HR), Denmark (DK), Finland (FI), Iceland (IS), Ireland (IE), Liechtenstein (LI), Luxembourg (LU), Norway (NO), Portugal (PT), Slovenia (SI), Sweden (SE), Switzerland (CH), The Netherlands (NL). Note that the axes have the same scale limits, although the visual projection could appear distorted

**Fig. 4:**
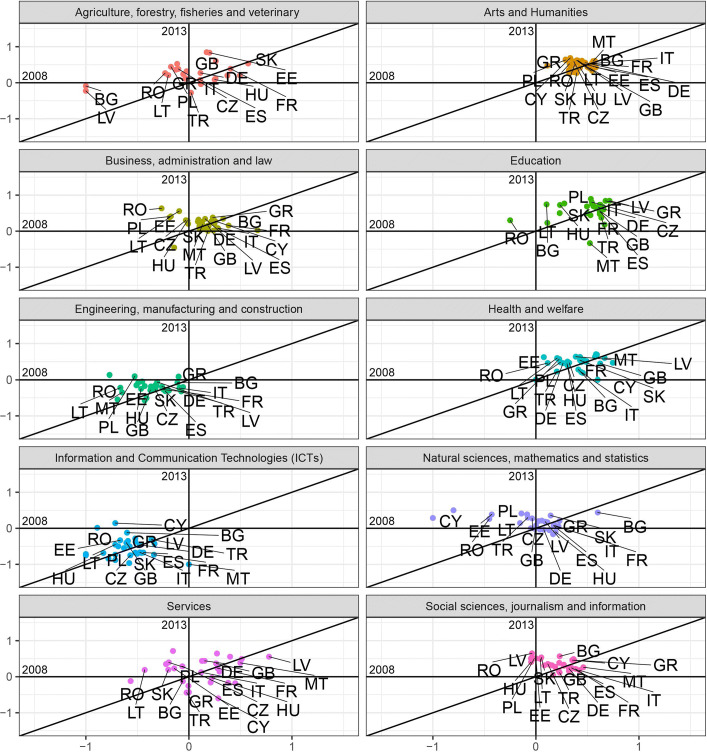
Gender balance in the outgoing flows across fields of study for the year 2008 against 2013. The scatter plots show the *F*/*M*^*B*^ measure for the outgoing flows of students for the years 2008 and 2013 for ten fields of study corresponding to the ISCED-F 2013 classification. Two macro fields (*Not known or unspecified* and *Generic programs and qualifications*) are not showed. The label of the following countries are not displayed to improve readability: Austria (AT), Belgium (BE), Croatia (HR), Denmark (DK), Finland (FI), Iceland (IS), Ireland (IE), Liechtenstein (LI), Luxembourg (LU), North Macedonia (MK), Norway (NO), Portugal (PT), Slovenia (SI), Sweden (SE), Switzerland (CH), The Netherlands (NL). Note that the axes have the same scale limits, although the visual projection could appear distorted

Differences between gender can be observed more in detail across fields of study. In this case, to obtain a clearer visualization, we use the symmetric transformation of the ratio *F*/*M* based on De Benedictis (2005), given by:

$$F/M^{B} = \frac{(F/M) - 1}{(F/M)+1}, $$

where the superscript *B* stands for *bounded*. The *F*/*M*^*B*^ index provides a measure of female participation over male participation with a value ranging (*bounded*) between [−1,1] and demarcation value equal to 0, corresponding to the absence of bias. Figures 3 and 4 plot this measure for 2008 against 2013 for each macro field of study in each country, respectively for incoming and outgoing students.

These plots allow to identify whether prevalence of one gender in a certain field has changed from 2008 to 2013. Briefly, for any field:

- country points located in the I quadrant (top-right) showed a female prevalence in 2008, that persisted in 2013 ($F/M_{2008,2013}^{B}>0$);
- country points located in the II quadrant (top-left) showed a male prevalence in 2008 ($F/M_{2008}^{B}<0$), that changed into female prevalence in 2013 ($F/M_{2013}^{B}>0$);
- country points located in the III quadrant (bottom-left) identify a higher male participation in 2008, that persisted in 2013 ($F/M_{2008,2013}^{B}<0$);
- country points located in the IV quadrant (bottom-right) signal a predominance of female students in 2008 ($F/M_{2008}^{B}>0$) that turned into a male prevalence in 2013 ($F/M_{2013}^{B}<0$).

Also, whether the cloud of points is positioned above or below the x-axis gives us an immediate picture of the prevalence of one gender over the other in 2013. In case of full-persistence all the dots would lay on the 45 ^∘^ line. The more they get disperse around the bisector the more variability from 2008 and 2013 is present (e.g. in Fig. 3 the Services field shows a high level of discontinuance in the gender ratio, while the Arts and Humanities field appears to be more persistent). The dots above the 45 ^∘^ line indicate an increase in the *F*/*M*^*B*^ ratio along time; the ones below indicate a decrease in *F*/*M*^*B*^.

At first glance, it is clear that there are two male-dominated sectors: Engineering, manufacturing and construction, and Information and Communication Technologies (ICTs). In the former, values for the incoming flows in 2008 show a higher variability than 2013, with a standard deviation *σ*_2008_=0.239 decreasing to *σ*_2013_=0.188 in 2013, and corresponding to values closer to the mean. For a few countries such as Bulgaria, Estonia, and Hungary the sector once dominated by female students in mobility changed tendency with a predominance of male students in 2013 (Fig. 3). The outgoing flows show more stability over time, except for Greece, Poland, and Romania, where the field once heavily dominated by outgoing male students had a reversed tendency in favor of female students in 2013 (Fig. 4). For the latter, the variability of the ratio also decreases from 2008 to 2013 considering the incoming flows, with Bulgaria representing the only case of reversed tendency in favor of females, while Cyprus and Greece had a change of the bias adjusting to the tendency of the other countries. On the contrary, in the outgoing flows variability increases during the time considered (*σ*_2008_=0.214 and *σ*_2013_=0.334), with mostly Eastern and some Mediterranean countries, such as Cyprus, outdistancing from the rest of countries towards more gender parity.

Looking at the overall fraction of female students in these two fields, without distinction of countries, allows for a few more considerations. Table 1 reveals that firstly, in absolute terms, the field of ICTs offers less mobility to students, probably as a reflection of the smaller number of students enrolled in this sector with respect to Engineering. Secondly, the share of women over males in Engineering is larger than in the ICTs, with a progress over the years analyzed, while not much changed in the ICTs. The greater distance from gender equality could, also in this case, simply be the result of the greater male intensity in terms of students enrolled in ICTs in Europe compared to Engineering.

**Table 1:** Number of female and male students, *F*/*M* ratio and *F*/*M*^*B*^ measure for the overall mobility in the male-intensive fields of study

		Female	Male	*F*/*M*	*F*/*M*^*B*^
Engineering, manufacturing and construction	2008	8799	15211	0.578	-0.267
	2013	12483	20202	0.618	-0.236
ICTs	2008	785	2752	0.285	-0.556
	2013	958	3218	0.298	-0.541

The rest of the sectors shows a predominance of female over male students, which in some cases remains pretty stable over the time span considered, as for Art and Humanities and Education, where the cloud of countries is concentrated around the bisector of the first quarter, with a few exceptions for the Education sector. Some Eastern countries (see Bulgaria, Romania, and Latvia for instance), in the incoming flows (Fig. 3), show a decrease of the female predominance from 2008 to 2013 and in the outgoing flows the extreme cases of Romania and Malta had a reverse of the index respectively in favor of men and women (Fig. 4).

In other cases, the values reported show less stability over time, as for the field of Health and welfare, both for the incoming and the outgoing flows, where volatility decreased from 2008 to 2013. Especially for the incoming flows, a group of Eastern countries (see Latvia, Poland, Slovakia) saw a decrease in the measure of bias thus in favor of a growing number of male students, up to cancel out the bias in the case of Romania.

The same observation can be drawn for the incoming flows of students in Business, administration and law, and Social sciences, journalism and information, where about the same group of Eastern countries reduces the gender bias over time in favor of greater male participation. In the outgoing flows, the behavior is opposite; starting with a bias in favor of male students, Eastern countries show to be catching up with the trend followed by the rest of countries.

Finally, in the fields of Agriculture, forestry, fisheries, veterinary and Services it is not possible to identify a general trend since the points displayed appear rather scattered, showing heterogeneity in the gender balance across countries; in the field of Natural sciences, Mathematics and Statistics, instead, the points appear concentrated around the mean value being close to the x-axis, with their position on the plane suggesting that the variance decreased over time and pointing out that gender disparities have been nearly cancelled, in a sector usually characterized by a larger male presence as common in the STEM-fields (Botella et al. 2019; OECD 2017).

**Fig. 5:**
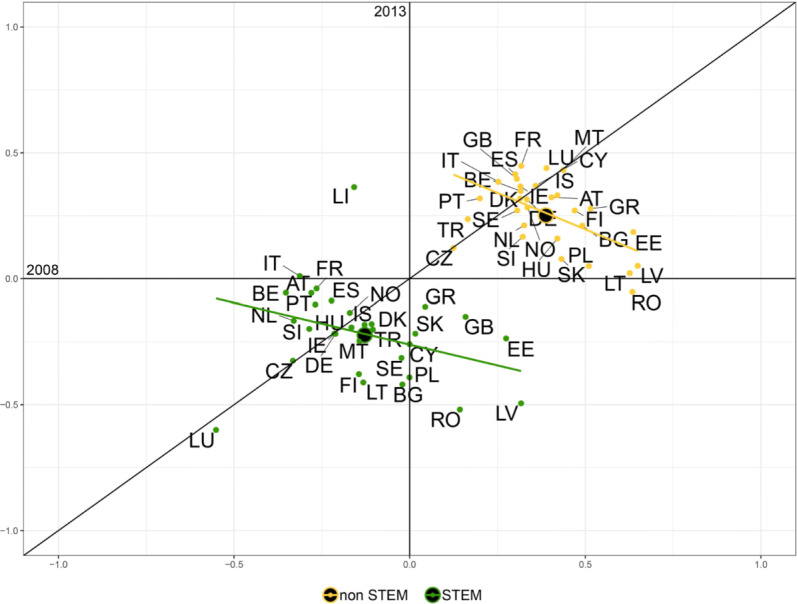
Gender balance in the incoming flows of students enrolled in STEM vs. non-STEM disciplines for the year 2008 against 2013. The scatter plot shows the *F*/*M*^*B*^ measure for the incoming flows of students for the years 2008 and 2013 by area of study. The STEM field is derived by summing the number of students in mobility in *Engineering, manufacturing and construction*, *ICTs* and *Natural sciences, mathematics and statistics*, as coded following the ISCED-F 2013 classification. Non-STEM disciplines include *Agriculture, forestry, fisheries and veterinary, Arts and Humanities, Business, administration and law, Education, Health and welfare, Services, Social sciences, journalism and information*. The fields *Not known or unspecified* and *Generic programs and qualifications* are not included in either the two groups. The fitted lines are robust to outliers and pass through the averages (black dot) of the two macro-fields

**Fig. 6:**
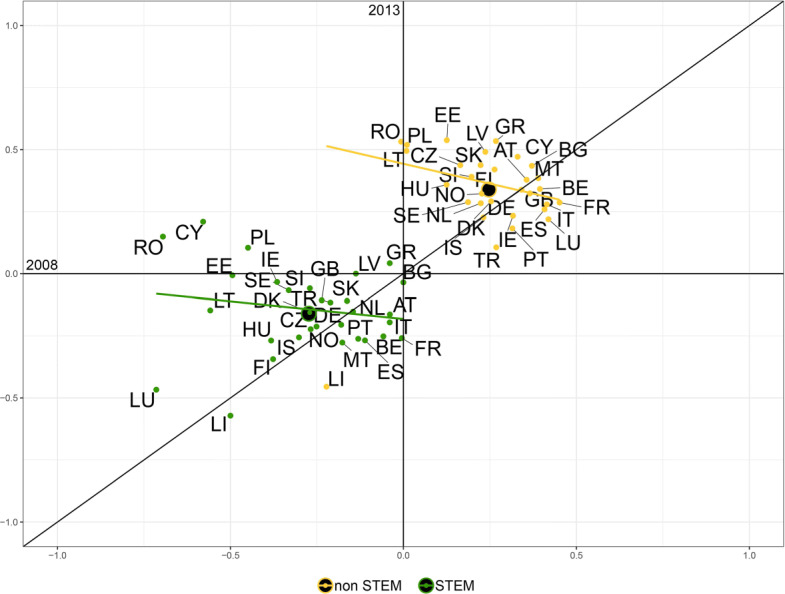
Gender balance in the outgoing flows of students enrolled in STEM vs. non-STEM disciplines for the year 2008 against 2013. The scatter plot shows the *F*/*M*^*B*^ measure for the outgoing flows of students for the years 2008 and 2013 by area of study. The STEM field is derived by summing the number of students in mobility in *Engineering, manufacturing and construction*, *ICTs* and *Natural sciences, mathematics and statistics*, as coded following the ISCED-F 2013 classification. Non-STEM disciplines include *Agriculture, forestry, fisheries and veterinary, Arts and Humanities, Business, administration and law, Education, Health and welfare, Services, Social sciences, journalism and information*. The fields *Not known or unspecified* and *Generic programs and qualifications* are not included in either the two groups. The fitted lines are robust to outliers and pass through the averages (black dot) of the two macro-fields

An even clearer pattern of gender differences by study area emerges, in fact, when considering STEM and non-STEM areas. In Figs. 5 and 6 the macro fields of study previously analyzed, as identified by the ISCED-F 2013 classification, are now grouped in STEM and non-STEM areas.

The two areas fully position themselves in opposite quadrants, highlighting a net difference in gender prevalence in these areas: STEM subjects are dominated by male students and, vice-versa, non-STEM fields show a female prevalence. This can be observed both for incoming and outgoing flows. The clouds of points are crossed by their respective interpolation lines resulting from a robust linear model, that are positioned oppositely to the displayed bisector. The slope of the lines signals a tendency towards convergence that suggests a trend towards a reduction of gender disparity in the incoming flows, since the average value is in both cases below the 45 ^∘^ line, and a reinforcement of the bias in outgoing flows.^10^ Considering the incoming students (Fig. 5), the group of countries below the bisector is numerous, both in STEM and non-STEM fields, and comprehends especially Eastern European countries. In the case of non-STEM subjects, this highlights that Eastern countries saw a decrease in the gender imbalance in favor of women, which in 2013 appears flattened to the zero, thus towards parity. In the STEM fields, the right wing of the group of dots still mainly represented by Eastern countries, as well as Greece and Great Britain, reports that for these countries there has been a reversal in the trend, from a female prevalence in 2008 to a male prevalence in 2013, consistent with what previously observed for the Engineering sector (see Fig. 3). For the outgoing flows, the countries position appears reversed, with a few countries (Romania, Cyprus, Poland) in STEM sectors changing gender prevalence from male in 2008 to female in 2013 (Fig. 6).

Briefly, decomposing the analysis over time by fields of study shows that Eastern and Mediterranean countries have seen a change in gender participation in the program, with a trend towards equality, as it could be generally observed in Fig. 2. The convergence toward the mean value of *F*/*M*^*B*^ is more pronounced in non-STEM fields, as the slope of the regression line shows for both incoming and outgoing student flows.

## The Erasmus network of universities

In this section we will visualize, describe and summarize the characteristics of the Erasmus program, comparing its structure in 2008, the initial year in our period, with 2013, the last year, keeping the resulting network of student flows separated by gender.

The Erasmus Network in the year *t*, $\mathcal {N}_{t}$, is a one-mode network defined by three sets: (a) the graph $\mathcal {G}_{t}=(\mathcal {V}_{t}, \mathcal {L}_{t})$: corresponding to the binary adjacency matrix, $\mathcal {A}_{t}$, and including the set of nodes (universities, belonging to different countries), $\mathcal {V}_{t}$, and the ordered set $\mathcal {L}_{t}$ of directed arcs (capturing the existence of an Erasmus exchange program between universities); (b) the edge value function, $\mathcal {W}_{t}$, containing, for every pair of nodes, the flows of students moving from the sending node to the receiving node, and the field of study; (c) and the node value function $\mathcal {O}_{t}$, containing information on universities, and on the country of activity. We examine the network in the two years *t*=2008;2013. In case of *t*=2008, $\mathcal {G}_{2008}=(\mathcal {V}_{2008}, \mathcal {L}_{2008})=(3148, 62221)$, while $\mathcal {G}_{2013}=(3148, 76446)$. From 2008 to 2013, 14225 more partnerships were established. The network appears to be characterized by a giant component, as found in Derzsi et al. (2011), and numerous isolated nodes; 861 are the total components that can be identified in 2008, while 497 in 2013. The giant component contains about 73% of all nodes in 2008 and grows to 84% of all nodes in 2013. The number of isolated nodes in 2008 was 858, while coherently with the increased dimension of the giant component, it decreased to 490 in 2013: 368 universities joined the Erasmus network in this time interval. The networks of the two reference years share 299 one-component elements among their isolated nodes. These nodes are represented by, presumably, small institutions, but certainly very specialized in their educational offer.^11^

Splitting the network according to gender, the network of the female flows in 2008 is $\mathcal {G}_{2008}^{F}=(\mathcal {V}_{2008}^{F}, \mathcal {L}_{2008}^{F}) = (3148, 47560)$, while the one of men is $\mathcal {G}_{2008}^{M} = (3148, 35558)$: the female Erasmus network is more connected than the male one. The simple comparison of the dynamics of $\mathcal {L}^{F}$ and $\mathcal {L}^{M}$ makes the first characteristic of the gender bias in the Erasmus Network evident: the ratio between $\mathcal {L}^{F}$ and $\mathcal {L}^{M}$ was 1.338 in 2008 ($\mathcal {L}_{2008}^{F}$ were 57.2% of total links, $\mathcal {L}_{2008}$, and $\mathcal {L}_{2008}^{M}$ were 75% of $\mathcal {L}_{2008}^{F}$). It is 1.329 in 2013 ($\mathcal {L}_{2013}^{F}$ were 57.1% of $\mathcal {L}_{2013}$, and $\mathcal {L}_{2013}^{M}$ were 72% of $\mathcal {L}_{2013}^{F}$). The values show a minimal variability during the years but, overall, the gender bias remains quite persistent.

Summary statistics of the female and male Erasmus network in 2008 and 2013 are summarized in Table 2.

**Table 2:** Summary statistics - Erasmus network - 2008 and 2013

	2008			2013		
	all	M	F	all	M	F
Active Universities	2290	2117	2209	2658	2444	2549
sending	2056	1893	1984	2356	2172	2233
receiving	2090	1873	1980	2419	2158	2295
University partnerships	62221			76446		
Active connections	83118	35558	47560	103160	44276	58863
Isolates	858	1031	939	490	704	599
Density	0.006	0.005	0.004	0.008	0.006	0.004
Degree	0.123	0.080	0.106	0.135	0.089	0.117
out	0.119	0.087	0.096	0.139	0.096	0.115
in	0.127	0.084	0.115	0.131	0.082	0.119
Closeness	0.00046	0.00043	0.00044	0.00054	0.00049	0.00051
out	0.00022	0.00018	0.0002	0.00022	0.00021	0.00024
in	0.0002	0.0002	0.0002	0.00022	0.00023	0.00022
Assortativity	-0.0068	-0.0125	0.0029	0.0086	0.0087	0.0212
Reciprocity	0.45	0.32	0.39	0.47	0.34	0.41
	GRA01	PRA07	GRA01	GRA01	GRA01	GRA01
	[395]	[286]	[318]	[463]	[315]	[380]
	MAD03	GRA01	MAD03	MAD03	MAD03	MAD03
	[354]	[274]	[304]	[413]	[279]	[351]
	BOL01	MAD03	WAR01	BOL01	BOL01	BOL01
Top-5 sending universities	[305]	[228]	[256]	[367]	[256]	[304]
	PRA07	BOL01	BOL01	VAL01	VAL01	VAL01
	[287]	[215]	[238]	[348]	[254]	[284]
	VAL02	SEV01	LJU01	LJU01	BAR03	LJU01
	[286]	[215]	[222]	[343]	[223]	[274]
	GRA01	VAL02	GRA01	GRA01	GRA01	GRA01
	[420]	[277]	[377]	[437]	[273]	[392]
	VAL02	GRA01	MAD03	MAD03	PRA07	MAD03
	[371]	[249]	[316]	[382]	[236]	[343]
	MAD03	MAD03	BOL01	BOL01	VAL02	BOL01
Top-5 receiving universities	[351]	[212]	[300]	[366]	[228]	[325]
	BOL01	VAL01	VAL01	VAL02	MAD03	VAL01
	[339]	[211]	[295]	[332]	[226]	[298]
	VAL01	LUN01	VAL02	LJU01	BOL01	BAR03
	[328]	[198]	[290]	[329]	[218]	[273]

**Note**: See Wasserman and Faust (1994) for the definition of the statistics used. M stands for male; F stands for female. Degree stands for Degree centralization (standardized); Closeness stands for Closeness centralization (standardized); Active con- nections includes student flows in different fields of study; Erasmus university codes have been shortened for visualization purpose: BAR03 = BARCELO03; BOL01 = BOLOGNA01; GRA01 = GRANADA01; LJU01 = LJUBLJA01; LUN01 = LUND01; MAD03 = MADRID03; PRA07 = PRAHA07; ROM01 = ROMA01; SEV01 = SEVILLA01; VAL01 = VALENCI01; VAL02 = VALENCI02; WAR01 = WARSZAW01. Squared parentheses contain the degree value. The Assortativity score is [-1,1]; the Reciprocity score is in percentage points

The overall density is rising, from a value of 0.006 to 0.008, giving evidence of a quite sparse network that is only moderately reducing its level of sparsity: the probability, for two universities chosen at random, to share an Erasmus program is 0.6% in 2008 and it becomes 0.8% in 2013.

The evolution of $\mathcal {N}_{t}^{F}$ and $\mathcal {N}_{t}^{M}$ from 2008 to 2013, made 340 (among the 939) isolated nodes in $\mathcal {G}_{2008}^{F}$ and 327 (among the 1031) isolated nodes in $\mathcal {G}_{2008}^{M}$ attached to the connected component of $\mathcal {N}_{2013}^{F}$ and $\mathcal {N}_{2013}^{M}$, respectively. The heterogeneity in connectivity is relevant and multifaceted: (1) the node with the highest *indegree* centrality, *v*_*max*_, had a deg(*v*_*max*_) equal to 377 for $\mathcal {G}_{2008}^{F}$ (it was the Universidad de Granada, in Spain), and to 277 for $\mathcal {G}_{2008}^{M}$ (the Universitat Politecnica de Valencia, in Spain); it becomes equal to 392 and 273 for $\mathcal {G}_{2013}^{F}$ and $\mathcal {G}_{2013}^{M}$, with the Universidad de Granada reaching the top position in both networks.^12^ (2) As far as *outdegree* centrality, the deg(*v*_*max*_) was equal to 318 for $\mathcal {G}_{2008}^{F}$ (it was the Universidad de Granada, in Spain), and to 286 for $\mathcal {G}_{2008}^{M}$ (the Univerzita Karlova of Prague, in the Czech Republic)^13^ and reached the values of 380 and 315 for $\mathcal {G}_{2013}^{F}$ and $\mathcal {G}_{2013}^{M}$, with the Universidad de Granada on top of both ranks.

To take the Universidad de Granada (E GRANADA01) as an example, in 2013, the University was sending female students to 380 foreign universities and was receiving international female students from 392 universities; with 268 universities the exchange of students was bilateral; with the remaining ones it was unilateral. In the same year, the University was sending male students to 315 foreign universities and was receiving international male students from 273 universities; with 173 universities the exchange of students was bilateral; with the remaining ones it was unilateral. 228 foreign universities were sending both female and male students to the Universidad de Granada; 164 foreign universities were sending only female students; 45 foreign universities were sending only male students. In the same year, the Universidad de Granada was sending both female and male students to 232 foreign universities, in the remaining 88 the University was sending only females, and to 83 was sending only males.

The Universidad de Granada example is paradigmatic: the amount of inter-gender flows is 50.1%, for university out-flows, and 52.2%, for university in-flows; the remaining flows are gender specific. Little bit less than half of the university flows are gender specific. In general, less than half of the universities tends to reciprocate, and, in 2013, reciprocity is more present among female flows (41%) than among male flows (34%).

Moving from ego to structural statistics, the Erasmus network is sparse (low density) and, at the same time, shows some relevant hubs, i.e. top sending universities, and authorities, i.e. top receiving universities (Kleinberg 1999),^14^ however, those hubs and authorities are connected to less than the 18% of the all active universities in the Erasmus network. There is no sign of a hierarchical structure: the closeness centralization is near zero and this implies that we are very far from a star-like structure. As previously emphasized, the whole network is composed of a set of communities of limited dimension. In the female Erasmus network, only 4.2% of the universities has more than 100 links and only the 34.2% has more than 10 links; in the male Erasmus network, only 2.3% of the universities has more than 100 links and only the 29.4% has more than 10 links. In 2008, the female and the male Erasmus networks were also different in terms of homophily: $\mathcal {G}_{2008}^{F}$ shows sign of mild assortativity, while $\mathcal {G}_{2008}^{M}$ shows sign of mild disassortativity: universities sending female students tend to have a positive match in terms of degree, in other therms, highly connected universities are connected with highly connected universities; universities sending male students tend, instead, to have a negative match, with highly connected universities being connected with poorly connected universities. This difference disappears in 2013, with both $\mathcal {G}_{2013}^{F}$ and $\mathcal {G}_{2013}^{M}$ showing a sign of mild assortativity.

$\mathcal {G}_{t}$ can also be explored in its weighted version $\mathcal {N}_{t}$, by accounting for the flow of students that each link has. By looking at the country level this time, the analysis allows understanding how, if on the one hand the number of participant countries has been stable in the last decade, as pictured in Fig. 1, the level of participation of each country is instead pretty heterogeneous, with student flows accounting for different proportion over the number of students enrolled at university. Table 3 shows the percentage in-strength and out-strength measures for each country, weighted by the flow of incoming and outgoing students, averaged over the years 2008–2013 and normalized to the total number of students enrolled in higher education in 2013.^15^ For the incoming flows, the in-strength measure highlights the role of countries such as Spain, France, Great Britain, Germany, and Italy as the destination for European university students; while for the outgoing flows, the out-strength draws attention to the role of small countries, such as Liechtenstein and Luxembourg, where a considerable part of the student population enrolled in higher education participates in the program.

**Table 3:** Average in-strength and out-strength for the years from 2008 to 2013, normalized respectively to the average number of students enrolled in higher education abroad in 2013 and the amount of students enrolled in higher education in the country of reference in 2013

	Average normalized in-strength	Average normalized out-strength
Austria	0.61	1.03
Belgium	0.80	1.16
Bulgaria	0.07	0.51
Croatia	0.05	0.40
Cyprus	0.05	0.71
Czech Republic	0.64	1.35
Denmark	0.74	0.73
Estonia	0.11	1.11
Finland	0.83	1.27
France	3.31	1.09
Germany	2.97	0.95
Great Britain	2.51	0.37
Greece	0.25	0.46
Hungary	0.41	0.93
Iceland	0.06	1.14
Ireland	0.57	0.91
Italy	2.34	1.07
Latvia	0.09	1.40
Liechtenstein	0.00	3.06
Lithuania	0.20	1.53
Luxembourg	0.01	6.52
Malta	0.06	1.03
Norway	0.49	0.57
Poland	1.07	0.62
Portugal	1.01	1.41
Republic of North Macedonia	-	0.15
Romania	0.18	0.54
Slovakia	0.14	1.01
Slovenia	0.18	1.27
Spain	4.28	1.54
Sweden	1.20	0.68
Switzerland	0.26	0.93
The Netherlands	1.05	0.90
Turkey	0.67	0.20

## Degree distribution analysis

By considering $\mathcal {G}_{t}$, the Graph of universities participating in the program, we analyze the degree distribution of this unweighted network by gender and along time, using 2008 and 2013 as initial and final benchmark years. Two universities are connected by a female-link (male-link) if there is at least one female (male) student moving from one university to the other one. As the network is directed, we observe both the indegree and the outdegree distribution. As common in most social networks, the degree distribution appears to be right-skewed in all cases. Tables 4 and 5 collect summary statistics for every distribution considered together with the estimated values of skewness and kurtosis. The degree distributions appear to be far from the normal or the exponential model, as instead observed in Derzsi et al. (2011), and thus they can be identified as “heavy-tailed” (Clementi 2016).

**Table 4:** Summary statistics and estimated skewness and kurtosis for the female indegree and outdegree distributions in 2008 and 2013

		Female						
		min	median	mean	max	estimated sd	estimated skewness	estimated kurtosis
Indegree	2008	0	2	18.86	669	46.26	5.81	53.03
	2013	0	4	23.57	668	52.15	4.81	37.01
Outdegree	2008	0	3	18.86	537	41.91	4.59	33.70
	2013	0	4	23.57	622	50.96	4.47	31.36

**Table 5:** Summary statistics and estimated skewness and kurtosis for the male indegree and outdegree distributions in 2008 and 2013

		Male						
		min	median	mean	max	estimated sd	estimated skewness	estimated kurtosis
Indegree	2008	0	2	13.63	403	32.09	4.96	37.79
	2013	0	3	16.98	392	36.19	4.19	27.06
Outdegree	2008	0	2	13.63	541	31.89	5.63	54.78
	2013	0	3	16.98	484	36.91	4.63	34.13

### Comparison along time

**Fig. 7:**
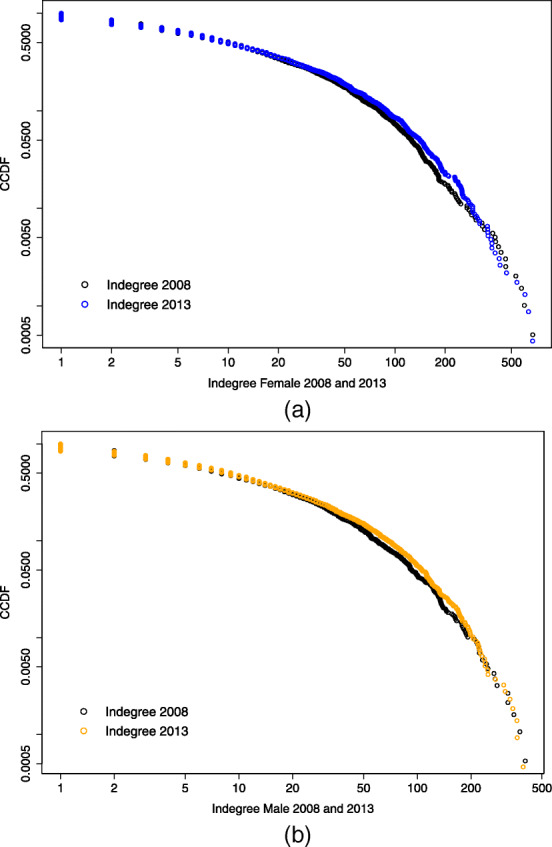
Female and male indegree distribution compared by year. Complementary cumulative distribution function (CCDF) on log-log scale of the female (a) and male (b) indegree distribution for the years 2008 and 2013

**Fig. 8:**
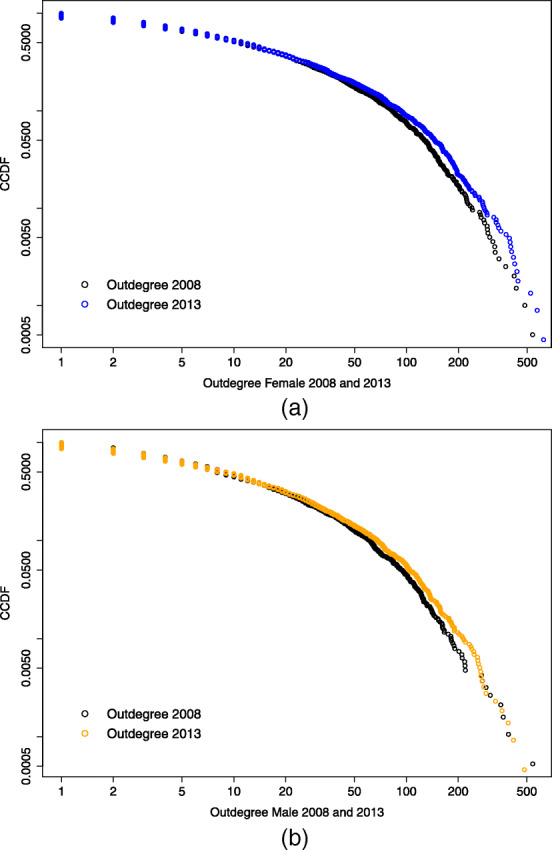
Female and male outdegree distribution compared by year. Complementary cumulative distribution function (CCDF) on log-log scale of the female (a) and male (b) outdegree distribution for the years 2008 and 2013

Figures 7 and 8 compare the complementary cumulative distribution function (CCDF), *Pr*(*X*)≥*x*, on a log-log scale respectively of the indegree and the outdegree for the years 2008 and 2013, for male and female links. Looking at the indegree distributions, both 2008 data are positioned below the 2013 and, especially for the female case, the CCDF corresponding to 2013 appears "fatter" in its central part, while the former looks more stretched. The tail of the distribution appears to have squeezed along the years considered. This is not visible for the outdegree distributions, which seem to have maintained the same shape along time.

### Comparison between genders

**Fig. 9:**
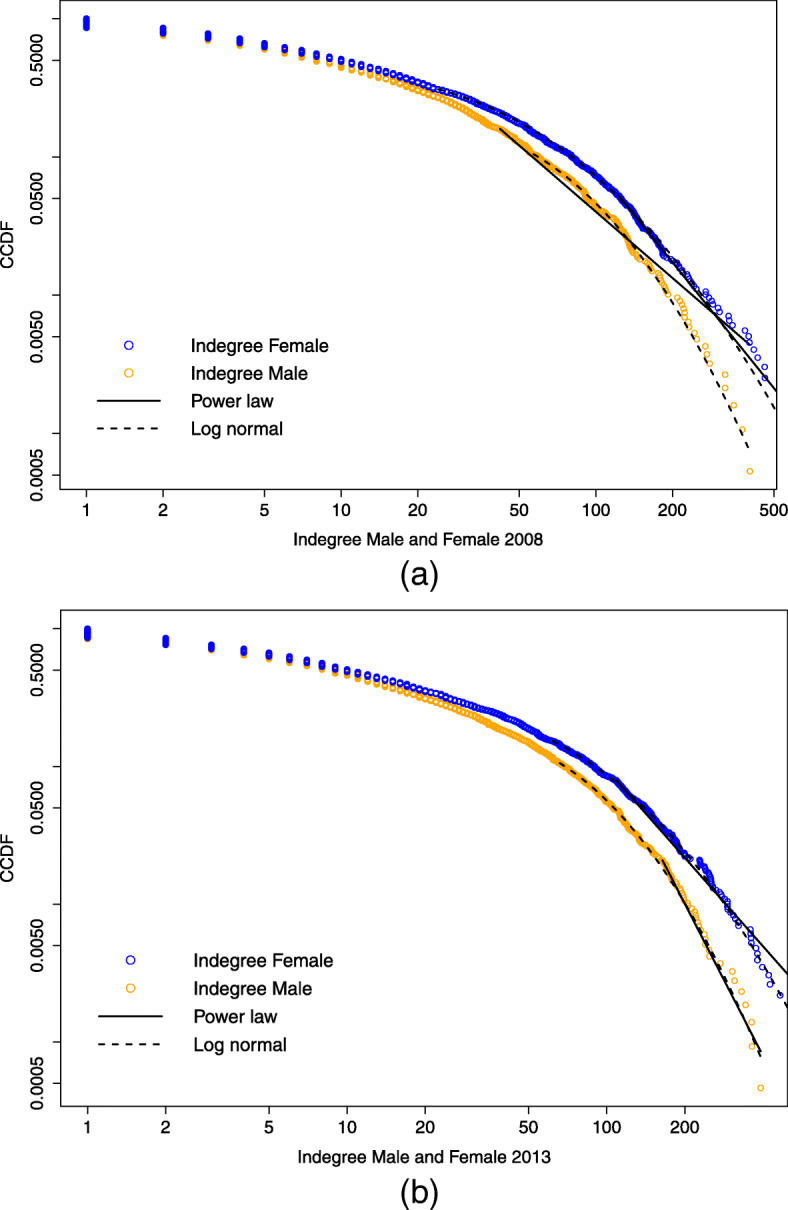
2008 and 2013 indegree distributions compared by gender. Complementary cumulative distribution function (CCDF) on log-log scale of the female and male indegree distribution for the years 2008 (a) and 2013 (b). For each empirical distribution, the plots display also the lines of fit for a power law and a lognormal distributional model

**Fig. 10:**
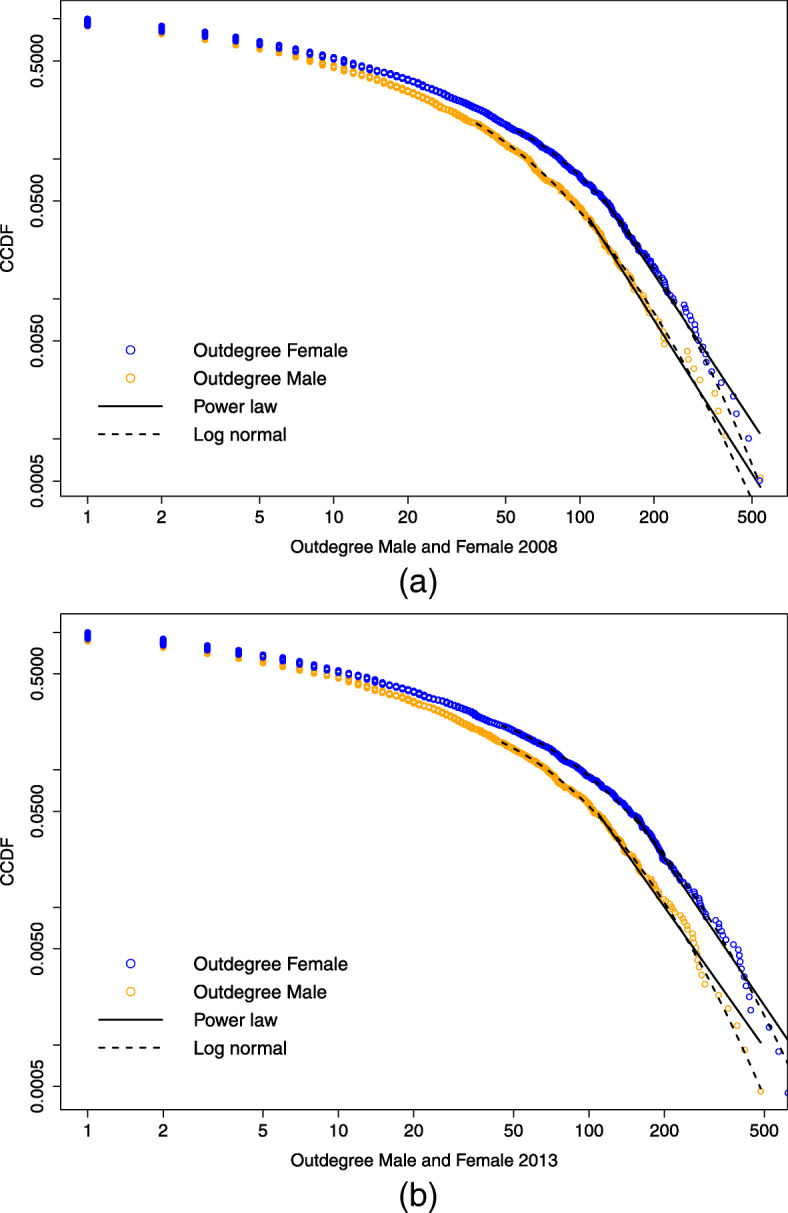
2008 and 2013 outdegree distributions compared by gender. Complementary cumulative distribution function (CCDF) on log-log scale of the female and male outdegree distribution for the years 2008 (a) and 2013 (b). For each empirical distribution, the plots display also the lines of fit for a power law and a lognormal distributional model

The same graphs are reported in Figs. 9 and 10, this time comparing the distribution for male and female connections in the same plot. The plotted densities include now also lines of fit for a power law distributional model and a lognormal distribution. A power law degree distribution, *p*(*x*)∝*x*^−*α*^, is observed in the so-called scale-free networks (Barabási et al. 1999), although the empirical distribution usually follows a power law model only in its upper tail, i.e. starting from a threshold *x*_*min*_.

The methodology employed for fitting heavy-tailed distributions is developed by Gillespie and et al. (2015) and based on Clauset et al. (2009); it relies on maximum likelihood estimators and goodness-of-fit based approach to determine the cut-off *x*_*min*_. In particular, the optimal choice of *x*_*min*_ is determined by minimizing the distance *D* between the probability distribution of the data and the best-fit power law model, measured by the Kolmogorov-Smirnov (K-S) statistic:

$$D = \max_{x\geq x_{min}} \mid S(x)-P(x)\mid, $$

where *S*(*x*) is the Cumulative Distribution Function (CDF) of the data and *P*(*x*) is the CDF for the power-law fitted model.

Following the framework proposed by Clauset et al. (2009), the visual inspection and the distribution fit are complemented with a goodness-of-fit test, based again on the K-S. Via bootstrapping, a distribution of the K-S statistic is generated following the creation of a large number of power-law distributed synthetic data sets with scaling parameter *α* and lower bound *x*_*min*_ equal to those of the distribution that best fits the observed data. A *p*-value is thus generated as the fraction of the time that the K-S statistic is larger than its value for the empirical data. The *p*-value quantifies the following hypotheses:

$$\begin{array}{l} H_{0}: \text{the power law fitted model is a plausible option} \\ H_{1}: \text{the power law fitted model is not a plausible option} \end{array} $$

Table 6 collects the estimated values for the scaling parameter *α* and the threshold value *x*_*min*_ for each power law fitted model and shows the relative K-S statistic together with the *p*-value resulting from the goodness-of-fit test for each case. As in Clauset et al. (2009) we decide to rule out the power law hypothesis if *p*≤0.1; this is the case for the female indegree distribution in 2013 and the male indegree distribution in 2008, which therefore are not well described by a power law.

**Table 6:** Estimated parameter and lower bound for a power law fitted model with relative K-S statistic and *p*-value for the goodness-of-fit test

		*x*_*min*_	*α*	K-S	*p*-value
Female	Indegree 2008	123	3.28	0.035	0.87
	Indegree2013	109	3.13	0.064	0.02
	Outdegree 2008	126	3.64	0.046	0.68
	Outdegree 2013	153	3.68	0.045	0.66
Male	Indegree 2008	42	2.59	0.073	0
	Indegree 2013	164	4.66	0.070	0.32
	Outdegree 2008	109	3.75	0.041	0.97
	Outdegree 2013	109	3.56	0.049	0.48

For the sake of accuracy, the analysis is complemented with a Vuong’s test comparing the power law fit with a lognormal distribution fit, which suggests that there is not a preferred model between those tested (Vuong 1989).

Although it is not trivial to identify the best distributional model for the degree distributions, some observations can be drawn. For the indegree distribution, we observe an opposite behavior along time in the tail of the female and male distribution. For the former the test conducted supports the hypothesis that the power law model is no longer a good description for the tail of this distribution. On the contrary, the male indegree distribution shows a “heavy” but not “fat” tail in 2008, meaning that its CCDF goes to zero faster than a power law; in 2013, instead, the tail of the male indegree distribution appears to be well described by a power law. This change in the distributional model might suggest that the top universities in terms of male connections are moving away from the body of the distribution, while the opposite is happening for the female, meaning that from 2008 to 2013 the universities with the most male links have been growing this kind of connection, while the universities with the most female connections are reducing their difference with the bulk of the distribution. Starting from a situation characterized by a strong gender imbalance in favor of female connections, this could signal a tendency towards increased gender parity in the Erasmus incoming connections.

On the other hand, the outdegree distribution remains pretty stable over time and does not behave much differently by gender. In each case considered, it seems well described by a power law model. We can hypothesize that concerning male students, whose degree distribution shows a scale-free behavior, when new universities adhere to the program, they follow a mechanism of preferential attachment: they connect with the most popular hubs, with high indegree (as well outdegree) values, not necessarily receiving a connection in the opposite direction, meaning that the outdegree value increases for those observations located in the core of the distribution, while the nodes with high outdegree measures do not move further away from the bulk of the distribution.

## Conclusions

The Erasmus Program has been characterized, since recently, by a strong gender bias in favor of female students. This work quantifies this evidence between 2008 and 2013, using novel data on bilateral student flows at the university level.

Key results can be summarized as follows. Firstly, after describing the structure of the program in great detail, a descriptive analysis across fields of study performed at country level has highlighted the prevalence of female students in mobility in the non-STEM disciplines, such as Arts and Humanities, Education, Business Administration and Law, and the opposite for the fields of study defined as STEM, such as Engineering and ICTs. It is also observed that moving from 2008 to 2013, there is an increasing trend toward gender parity, especially in Eastern European and some Mediterranean countries.

Secondly, the study of the participation of the 3148 universities included in the program between 2008 and 2013 through network analysis emphasized that in general the gender bias persisted over time, given the denser network of connections involving female students. In addition, the female Erasmus network is characterized by a higher level of reciprocity and homophily, with also a prevalence of sender universities rather than receivers, contrary to the male Erasmus network. The Universidad de Granada in Spain stands out as key sender for the female network, in both years analyzed, and in 2013 replaced the Univerzita Karlova of Prague in its role of key sender in the male network in 2008. The Universidad de Granada is also the key receiver in 2013 for both the female and male network, replacing the Universitat Politecnica de Valencia, in Spain as key receiver in 2008 for the male network. The network of universities is characterized by a giant component including the majority of nodes.

Finally, the paper also tests the difference in the degree distribution of the Erasmus network along time and between gender, giving evidence of contrast with respect to the exponential behavior resulting from (Derzsi et al. 2011) for 2003 and a variation along time of the indegree distribution. Considering the female network of universities, the tail of its degree distribution follows a power law model in 2008 while a lognormal distribution could better describe it in 2013; the opposite behavior characterizes the indegree distribution in the male network of universities, signaling a tendency towards balancing the initial strong gap in the incoming connections.

In conclusion, evidence from this work underlines a mild trend towards the reduction of the strong gender bias in favor of female students that characterized Erasmus in 2008 and suggests that this tendency can foster the convergence of male and female students flows possibly resulting in a future annulment or at least considerable reduction of the bias. The availability of detailed data at the university level on bilateral flows of Erasmus students for more recent years will make possible to confirm or deny this early evidence.

From a normative point of view, the effect of the observed gender imbalance might be twofold. On the one hand, the bias in favor of female students may have a positive impact on women empowerment, since international experiences could facilitate entry into the labor market, which is known to be *ceteris paribus* more difficult for women. On the other hand, considering that it is likely that men, in particular, will lead the labor markets in the future, enlarging their international experience could increase their skills and promote an attitude towards international benchmarking in decision making. It is the responsibility of policymakers to understand whether gender equality would be desirable in Erasmus student mobility and, consequently, adopt the most appropriate policy tools to orient student flows.

The richness of the Erasmus data and relevance of the analysis on international formation of human capital call for future research. The time dimension could be analyzed in more detail by checking for possible differences and similarities across the period, not only comparing each year included but adopting a dynamic approach to the network data (Batagelj et al. 2014). A further extension of the present work could be the adoption of a blockmodeling approach, along the lines of (Restaino et al. 2020), to highlight the presence of sub-networks and communities in the network of universities. The longitudinal data could be also exploited in a more model-based analysis. Using a gravity framework (Monte et al. 2018) could help understand the relationship between the bilateral flow of Erasmus students and the country and university characteristics both as sender and receiver, and to explore if the distance elasticity has a gender and/or a field dimension. This analysis could be complemented by the inclusion of economic indicators and information about higher education at country level provided by EUROSTAT or at the university level, using international quality ranks of universities.

In general, the growth of the Erasmus program in terms of student participation suggests that it will continue to grow, considering also the increased funding devoted to the initiative. Conversely, the number of participating countries is rather stable, but this reflects the political dynamics of EU Member and partner countries. For instance, the recent exit of Great Britain from the European Union raises doubts about possible consequences for the Erasmus program, which necessarily requires stable agreements between universities to allow study abroad without incurring in the payment of tuition fees in the host country. Future research could explore possible effects that are still uncertain today. Finally, the recent COVID-19 pandemic might have changed the future of the Erasmus program with universities in lockdown and courses entirely provided online. The success of the Erasmus experience relies on the active participation of students in university life in the host country, which includes using the university facilities, relating with professors and classmates using a foreign language, and experiencing the everyday life typical of the host country. What will be of this exchange program with the conditions imposed by the pandemic is still unknown. Future research could shed light on possible scenarios as well as on the potential choice of students to postpone or give up the Erasmus experience.

## Data Availability

The original datasets used during the current study are available in the EU open data portal repository, [https://data.europa.eu/euodp/en/home]. The datasets generated by the authors, R scripts and Stata do files are available on request for replication purposes.

## Abbreviations

CCDF: Complementary Cumulative Distribution Function
CDF: Cumulative Distribution Function
ERASMUS: EuRopean community Action Scheme for the Mobility of University Students
ICTs: Information and Communication Technologies
ISCED: International Standard Classification of Education
STEM: Science, Technology, Engineering and Mathematics

## Footnotes

1 The Erasmus program includes also a variety of other initiatives, such as volunteering activities, Erasmus Mundus Joint Master Degrees, the European Week of Sports, Jean Monnet Modules and Chairs.

2 See Souto-Otero et al. (2013) for an investigation of the barriers to the Erasmus program.

3 In this cross-sectional analysis, Bottcher et al. (2016) specifically study the over-representation of female Erasmus students by comparing tertiary education statistics with the Erasmus data using a null model for which it is assumed that the population of Erasmus students is randomly drawn from the student population of participating countries.

4 The development of emerging economies and women’s emancipation in developing countries also play a role, as in the case of China, which saw an economic boom along with a change in the role of women, who became more individually-oriented and delayed their marriage age (Martin 2014).

5 Science, Technology, Engineering and Mathematics.

6 International Standard Classification of Education.

7 The name of the program comes from the Latinisation of the name of Erasmus of Rotterdam. The success of this program has led to the use of naming other European programs after famous personalities from various European cultures such as Socrates, Leonardo or Comenius (Corradi 2015).

8 Erasmus+ is, in fact, part of a socio-economic context that, on the one hand, counts more than 3 million young Europeans unemployed in 2019, with youth unemployment rates reaching 30% in some countries, and on the other hand, almost 4 million job vacancies, with more than a third of employers reporting difficulties in recruiting staff with the required qualifications (European Commission 2018).

9 This is the case of Austria, Finland and Sweden, for example, which became EU Member States in 1995 (as shown in Fig. 1), but joined the Erasmus program as early as 1992; or the case of those countries that joined the EU in 2004 but have been participating in Erasmus since 1998 and 1999.

10 A robust (to outliers) linear model has been estimated also controlling for the student population in higher education in the countries considered, however, it showed no significant change with respect to the unconditioned bivariate model displayed in Figs. 5 and 6.

11 Some examples are the Stockholms Musikpedagogiska Institut, the University College of Music Education in Stockholm, specialized in teaching music and related arts, or the Shipping and Transport College Group based in Rotterdam and providing maritime transport and logistics education. Among these components we find also (private) foundations with a religious nature (e.g. Association Familiale de l’externat Sainte-Marie, based in Lyon) or institutions providing secondary education in the form of two-years preparation for admission to a university, usually following the compulsory stage of secondary education (known as Bachillerato in Spain, A levels in the UK, Baccalauréat in France).

12 The Universidad de Granada (E GRANADA01) receives students from 70 German universities; 65 French universities; 62 Italian universities; 39 universities from the UK; 24 from Poland; 15 from Belgium; 11 universities from Turkey, Portugal, Greece, and the Netherlands; 10 from Austria and less then 63 universities from the remaining countries; with a total 392 universities (in 2013) sending students, males and females, in all fields of study.

13 In 2008, the Univerzita Karlova of Prague (PRAHA07) was sending male students to 52 French universities; 41 German universities; 33 British universities; 27 Spanish universities; 18 Italian universities; 14 universities of Finland; 13 of Sweden; 10 of Portugal and Austria; and 68 other universities of the other countries of the Erasmus program.

14 According to Kleinberg (1999), the authority scores of the vertices of a Graph are defined as the principal eigenvector of $t(\mathcal {A}) \cdot \mathcal {A}$, where $\mathcal {A}$ is the adjacency matrix of the Graph. The hub scores are the principal eigenvector of $\mathcal {A} \cdot t(\mathcal {A})$. The University of Bologna (I BOLOGNA01) is the major hub in 2008 and the distribution of the hub scores is power law: the large majority of universities are poorly connected. As far as the authority score, the Universidad de Granada (E GRANADA01) gets the highest value, and also in the case of the authority score the distribution is power law. If we look at the difference between the male and female Erasmus network the comparison between hubs and authorities distributions gets inverted: the distribution of the authority score decreases sharply before the one of the hub score in the female network, the opposite happens in the male network. The gap gets reduced in 2013. However, the difference between the female and male network in terms of the prevalence of sending universities over receiving universities, for females, and of receiving universities over sending universities, for males, persists. More about this in the next paragraph.

15 In the case of countries that joined the Erasmus program later than 2008, the average is taken only on the years of participation.
